# Primary high-grade serous ovarian cancer cells are sensitive to senescence induced by carboplatin and paclitaxel in vitro

**DOI:** 10.1186/s11658-021-00287-4

**Published:** 2021-10-21

**Authors:** Paweł Uruski, Agnieszka Sepetowska, Corinna Konieczna, Martyna Pakuła, Michał Wyrwa, Akylbek Tussupkaliyev, Andrzej Tykarski, Justyna Mikuła-Pietrasik, Krzysztof Książek

**Affiliations:** 1 Department of Hypertensiology, Poznań University of Medical Sciences, 1/2 Długa St., 61-848 Poznań, Poland; 2 Department of Pathophysiology of Ageing and Civilization Diseases, Poznań University of Medical Sciences, 1/2 Długa St., 61-848 Poznań, Poland; 3 Department of Obstetrics and Gynecology, West Kazakhstan Marat Ospanov Medical University, 50B 12th Microdistrict Apt. 21, 030008 Aktobe, Republic of Kazakhstan

**Keywords:** Cellular senescence, Drug-induced senescence, Ovarian cancer, Senescence-associated secretory phenotype

## Abstract

**Background:**

Various types of normal and cancer cells undergo senescence in response to carboplatin and paclitaxel, which are considered the gold standard treatments in ovarian cancer management. Surprisingly, the effect of these drugs on ovarian cancer cell senescence remained unknown.

**Methods:**

The experiments were conducted on primary high-grade serous ovarian cancer cells. Molecular markers of senescence were evaluated using cytochemistry and immunofluorescence. Cell cycle distribution was analyzed using flow cytometry. Expression of cyclins and signaling pathways was tested using western blot. Telomere length and telomerase activity were measured using qPCR, and the colocalization of telomeres with DNA damage foci using immuno-FISH. Oxidative stress-related parameters were quantified using appropriate fluorescence probes. Production of cancerogenic agents was analyzed using qPCR and ELISA.

**Results:**

Carboplatin applied with paclitaxel induces senescence of ovarian cancer cells in vitro. This activity was reflected by permanent G2/M growth arrest, a high fraction of cells expressing senescence biomarkers (SA-β-Gal and γ-H2A.X), upregulated expression of p16, p21, and p53 cell cycle inhibitors, and decreased expression of cyclin B1. Neither telomere length nor telomerase activity changed in the senescent cells, and the majority of DNA damage was localized outside telomeres. Moreover, drug-treated cancer cells exhibited increased production of STAT3 protein, overproduced superoxide and peroxides, and increased mitochondrial mass. They were also characterized by upregulated ANG1, CCL11, IL-6, PDGF-D, TIMP-3, TSP-1, and TGF-β1 at the mRNA and/or protein level.

**Conclusions:**

Our findings imply that conventional chemotherapy may elicit senescence in ovarian cancer cells, which may translate to the development of a cancer-promoting phenotype, despite the inability of these cells to divide.

**Supplementary Information:**

The online version contains supplementary material available at 10.1186/s11658-021-00287-4.

## Background

Although cellular senescence is a postmitotic state in which cells irreversibly lose the ability to divide and is classically considered the ultimate fate of normal cells bearing extensive and irreparably deteriorated DNA [1, 2], in recent years, the view that senescence may also be elicited in cancer cells subjected to radio- and chemotherapy has been increasingly supported [3]. The list of agents capable of triggering drug-induced premature senescence (DIPS) of cancer cells is long and includes aphidicolin, bleomycin, cisplatin, doxorubicin, etoposide, mitoxantrone, retinols, hydroxyurea, carboplatin combined with docetaxel, and many others [4]. The senescence-promoting activity of conventional chemotherapeutics has been documented in several tumors, such as breast, lung, prostate, and colon carcinoma [4], irrespective of their p53 profile [5].

Among various female genital tract malignancies, epithelial ovarian cancer is the most lethal [6]. Because the majority of ovarian cancer cases are diagnosed at advanced stages of the disease, patients are recommended to undergo maximal surgical debulking followed by systemic administration of carboplatin (CPT) and paclitaxel (PCT), which is considered the best chemotherapeutic option [7]. Despite different modes of action, both drugs have been found to jeopardize normal cell functioning, leaving a mark on their energetic metabolism, driving their inflammatory responses and transition to the reactive stroma, generating DNA injury, and inducing apoptosis and senescence [8, 9]. Surprisingly, the potential impact of CPT and/or PCT on primary ovarian cancer cell senescence has never been systematically tested either in vitro or in vivo. Taking this into account, we designed a study in which the effect of a CPT-PCT mixture applied at clinically relevant doses [10] to a wide range of molecular senescence-associated parameters in cultured primary high-grade serous ovarian cancer cells (HGSOCs) was examined and discussed.

## Materials and methods

### Cell cultures and experimental conditions

Primary high-grade serous ovarian cancer cells (HGSOCs) were isolated from tumors obtained from 14 different patients during cytoreductive surgery. The tissues were cut into small fragments of similar weight and then placed in 0.05% trypsin-0.02% EDTA for 20 min at 37 °C with shaking. The cells were grown in RPMI 1640 supplemented with l-glutamine (2 mM) and 20% FBS. Their cancerous nature was confirmed according to the expression of epithelium-related antigen (MOC-31) and CA125 antigen. The study was approved by an institutional ethics committee (consent number 578/18), and all patients gave their informed consent.

Cells from the first passage (young cells) were incubated with 50 µM carboplatin (CPT) combined with 25 nM paclitaxel (PCT) (both from Cayman Chemical, Ann Arbor, MI, USA) in standard growth medium for 72 h at 37 °C. Then, the cells were rinsed with Hanks’ balanced salt solution and incubated in standard growth medium for 24 h to recover. Then, the culture was passaged and incubated in standard conditions for an additional 7–10 days. After incubation, the cells were considered senescent, which was confirmed by their morphology, inability to proliferate, and the presence of senescence biomarkers. The concentrations of drugs were established in pilot experiments on the basis of clinical observations [10] and experimental data [11]. The time of exposure was selected according to our pilot studies which allowed us to identify the length of treatment which does not induce massive cell death but results instead in certain metabolic effects associated with cellular senescence.

### Detection of senescence biomarkers

The expression of senescence-associated β-galactosidase (SA-β-Gal) in cell cultures was visualized as described previously [12]. Immunofluorescence of damage response (DDR) elements, i.e., the foci of the phosphorylated variant of histone H2A.X (γ-H2A.X) and p53-binding protein 1 (53BP1), was tested as described previously [13] using anti-γ-H2A.X (cat #ab2893, Abcam, Cambridge, UK) and anti-53BP1 antibodies (cat #NB100-304, Novus Biologicals, Abingdon, UK). In some experiments, the detection of γ-H2A.X was followed by the visualization of telomeric ends using the Telomere PNA FISH Kit/Cy3 (Dako, Carpinteria, CA, USA) according to the manufacturer’s instructions. To verify the colocalization of γ-H2A.X foci with telomeres, deconvoluted images were examined using the Costes approximation methodology with ImageJ v1.53e software (http://rsb.info.nih.gov/ij/), enriched in the plugins obtained from the Wright Cell Imaging Facility (http://www.uhnresearch.ca/facilities/wcif/imagej/). By moving from left to right and from top to bottom 500 cells (SA-β-Gal, γ-H2A.X, 53BP1) or nuclei (colocalization) were analyzed.

### Analysis of cell cycle distribution and inhibitory proteins

The distribution of cells (10 000) in the cell cycle was determined using flow cytometry as described previously [14]. The fraction of cells expressing p16, p21, and p53 cell cycle inhibitors was quantified using immunofluorescence. Cells were fixed with 4% paraformaldehyde and 100% methanol, permeabilized with 0.1% Triton-X in PBS for 10 min, and blocked with a solution containing 1% bovine serum albumin, 22.5 mg/mL glycine, and 0.1% Triton-X in PBS for 30 min. Afterwards, specimens were incubated with antibodies directed against p16 (#ab108349; Abcam), p21 (#2947, Cell Signaling Technology, Danvers, MA, USA), and p53 (#2527, Cell Signaling), prepared at 1:200 dilution, for 24 h, at 4 °C. Then, the cells were extensively washed and subjected to DyLight 488 IgG (#ab96899, Abcam; 1:500) for 1.5 h at room temperature. After the incubation, the specimens were preserved with Fluoroshield medium with DAPI (Abcam) and inspected under a fluorescence microscope. By moving from left to right and from top to bottom, 500 cells were analyzed.

### Quantification of telomeres and telomerase

Telomeres were quantified using an Absolute Human Telomere Length Quantification qPCR Assay Kit (ScienCell, Carlsbad, CA, USA), whereas telomerase activity was measured using a TRAPEZE XL Telomerase Detection Kit (Merck). Both the assays were performed strictly according to manufacturers’ instructions.

### Examination of cyclins and signaling molecules

The expression of arbitrarily selected cyclins, kinases, and transcription factors was investigated using western blot. After SDS-PAGE, the proteins were transferred to a PVDF membrane (Serva, Heidelberg, Germany) using the Mini Trans Blot Module (Bio-Rad, Hercules, CA, USA; 70 V for 2 h). The membranes were then incubated with antibodies against cyclin B1 (#12,231), cyclin D1 (#2978), AKT (#9272), NF-κB p65 (#8242), p38 MAPK (#9212), p44/42 MAPK Erk1/2 (#4695), and STAT3 (#12,640) (all from Cell Signaling Technology, 1:1000) and a specific primary rabbit antibody against GAPDH as the control (Cell Signaling Technology, 1:500). Target proteins were visualized after incubation with peroxidase-labeled secondary antibodies (Cell Signaling), diluted 1:10,000, for 1 h at room temperature, followed by treatment with Immobilon Classico Western HRP substrate (Merck Millipore, Burlington, MA, USA) for 5 min at room temperature in the dark. The bands were analyzed using Image Lab software (Bio-Rad).

### Measurements of oxidative stress

The formation of reactive oxygen species (superoxide and peroxides) was monitored in cells stained with MitoSOX Red (Thermo Fisher Scientific) and dihydrorhodamine 123 (DHR), respectively. Changes in mitochondrial inner membrane potential (ΔΨ_m_) were determined in cells treated with 5,5ʹ,6,6ʹ-tetrachloro-1,1ʹ,3,3ʹ-tetraethylbenzimidazolylcarbocyanine iodide (JC-1; Cayman Chemical). Mitochondrial mass was estimated in cells incubated with 10-*n*-nonyl-acridine orange (NAO). All measurements were conducted as described previously [15].

### Analysis of the senescence-associated secretory phenotype (SASP)

Total RNA was isolated using TRI Reagent (Merck) and then purified using a Clean-up RNA Concentrator kit (A&A Biotechnology, Gdynia, Poland). The concentration of RNA was estimated according to an optical density at 260 nm. Its purity was determined by a 260/280 nm absorption ratio (> 1.8). Synthesis of cDNA was performed using the GoScript Reverse Transcription System (Promega Corporation, Madison, WI, USA). Real-time PCR was carried out using PowerUp SYBR Green Master Mix (Thermo Fisher Scientific) and appropriate primers (Merck). All reactions were performed on an Applied Biosystems 7500 Real-Time PCR System thermocycler (Thermo Fisher Scientific) under the following conditions: 2 min at 50 °C, 2 min at 95 °C, 45 cycles of 15 s at 95 °C, and 1 min at 60 °C. The quality of each amplicon was evaluated on the basis of the course of dissociation curves obtained via Melt Curve Stage at the end of each PCR. The relative expression levels were determined according to the 2^−ΔΔCt^ method with the glyceraldehyde-3-phosphate dehydrogenase (*GAPDH*) gene as the reference. Sequences of primers used during the RT-qPCR are presented in Table 1.

**Table 1:** Sequences of primers used during real-time PCR reactions

Gene	Forward	Reverse
ANG1	GCCTACACTTTCATTCTTCCAGA	TCTTCCTTGTGTTTTCCTTCCAT
CCL11	CCCTTCAGCGACTAGAGAGC	CAGCTTTCTGGGGACATTTG
CXCL12	GTGCCCTTCAGATTGTAGCC	CCTTCCCTAACACTGGTTTCA
IL-6	AAAGAGGCACTGGCAGAAAA	AGCTCTGGCTTGTTCCTCAC
PDGF-D	CCCAGGAATTACTCGGTCAA	ACAGCCACAATTTCCTCCAC
TIMP3	CTTCCAAGAACGAGTGTCT	GGTCTGTGGCATTGATGA
tPA	GACGTGGGAGTACTGTGATGTG	CCCTCCTTTGATGCGAAACTGA
TSP-1	GGCAAGGACTGCGTTGGT	CACTTCACGCCGGCAAAG
TGF-β1	CAGCAACAATTCCTGGCGATACC	GCGCTAAGGCGAAAGCCCTCAAT
VEGF	ATCTTCAAGCCATCCTGTGTGC	GCTCACCGCCTCGGCTTGT
GAPDH*	TCAAGATCATCAGCAATGCC	CGATACCAAAGTTGTCATGGA

^*^GAPDH was used as the reference gene

To evaluate the cell secretome, conditioned media were collected from young and senescent HGSOCs, as described previously [16]. Proteins were quantified using the appropriate DuoSet Immunoassay Development kits (R&D Systems, Abingdon, UK).

### Statistics

Statistical tests were conducted using GraphPad Prism 6.00 (GraphPad Software, San Diego, USA). The means were compared using the Wilcoxon test. The data are shown as the means ± SEM. Differences with a *p*-value less than 0.05 were considered significant.

## Results

### CPT-PCT combination induces senescence in primary HGSOCs

**Fig. 1:**
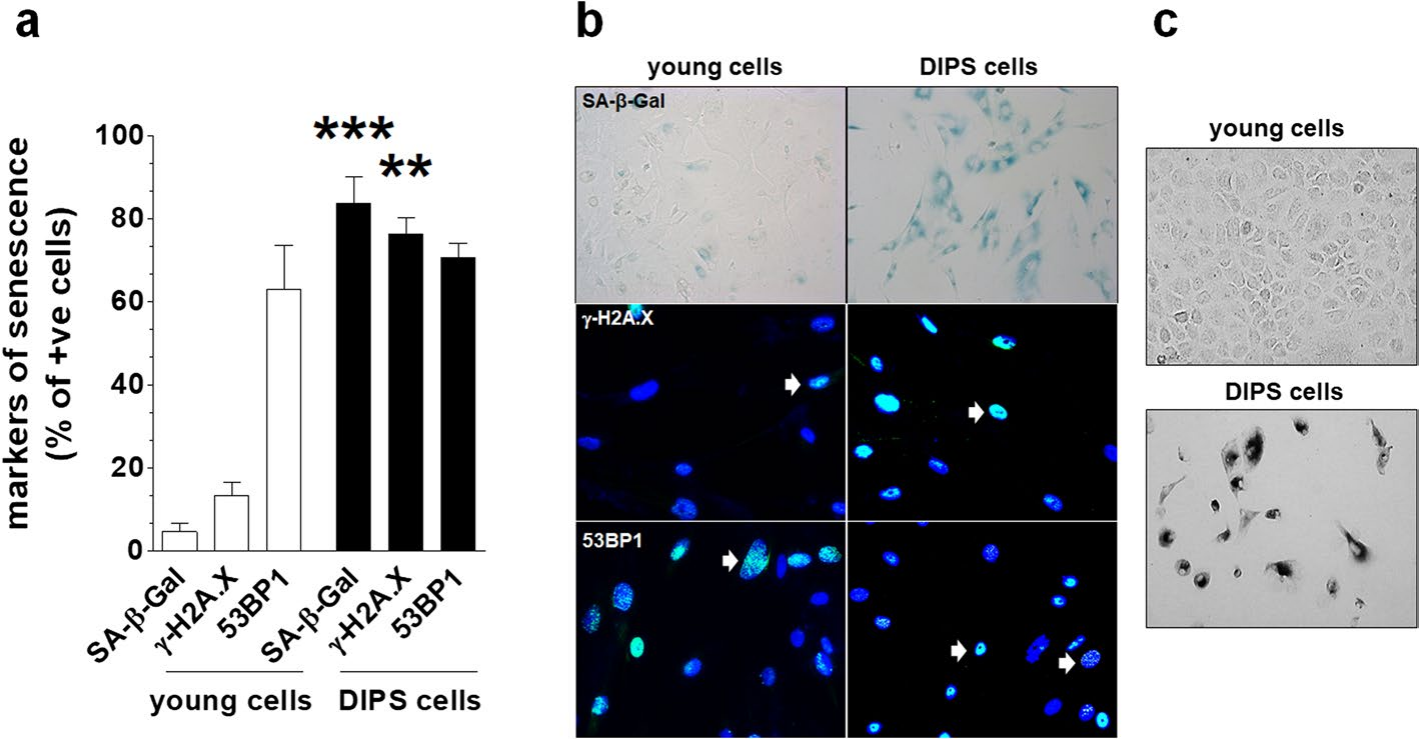
Development of the senescence phenotype in HGSOCs treated with CPT-PCT. For details regarding the procedure of CPT-PCT-dependent senescence induction please refer to “Methods”. Quantification of the fraction of cells displaying biochemical (SA-β-Gal) and molecular (γ-H2A.X, and 53 BP1) markers of cellular senescence in young and DIPS cancer cells (**a**). Representative staining against senescence biomarkers. SA-β-Gal-positive cells are characterized by green cytoplasm. White arrows indicate exemplary γ-H2A.X- and 53BP1- positive cells (**b**). Representative illustrations of young and DIPS cells’ appearance obtained upon changing the color image to black and white, sharpening and increasing contrast in cells stained for SA-β-Gal (**c**). Magnification × 200, objective aperture 0.35. The results are based on 6–8 independent experiments using HGSOCs obtained from different patients. The results are expressed as the means ± SEM. **p < 0.01; ***p < 0.001 vs. young cells

Primary HGSOCs isolated from tumors from chemotherapy-naïve patients were exposed to CPT (50 µM) combined with PCT (25 nM) to verify their sensitivity to drug-induced premature senescence (DIPS). Two kinds of senescent biomarkers were analyzed: the activity of SA-β-Gal and the activation of the DNA damage response marked by the presence of γ-H2A.X and 53BP1 foci. The experiments showed that first passage cell cultures undergoing DIPS permanently lost the ability to proliferate, which was manifested by their inability to reinitiate division for three weeks. At the same time, they developed hypertrophic morphology (Fig. 1c), and the majority of them displayed activity of SA-β-Gal and the presence of γ-H2A.X foci. Notably, the fraction of SA-β-Gal- and γ-H2A.X-positive cells increased significantly during senescence, as in the young, early-passage cells the percentage of cells bearing the senescence signature was far below 20%. Conversely, the fraction of 53BP1-positive cells was unexpectedly high in young cells and did not change any further upon treatment with CPT/PCT (Fig. 1a–c).

### Characteristics of DIPS-associated cell cycle arrest

**Fig. 2:**
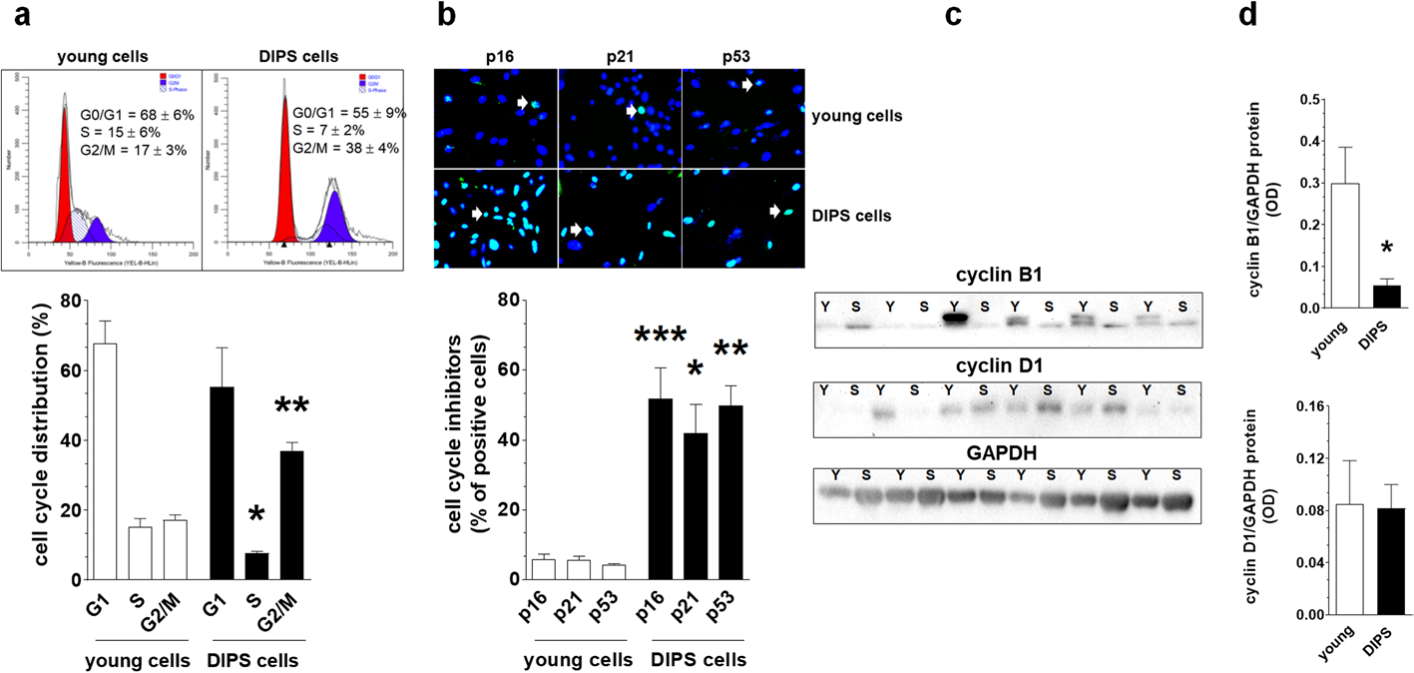
Characteristics of DIPS-associated growth arrest according to cell distribution in the cell cycle and the analysis of cell cycle inhibitory proteins and cell cycle-promoting cyclins. Analysis of cell distribution in particular phases of mitotic cycle using flow cytometry with cells stained with propidium iodide. The cells gathered in the G1 phase are marked in red, whereas cells belonging to the G2/M stage are marked in blue. The quantification (%) of cells arrested in G0/G1, S, and G2/M phases is shown on the representative histograms (**a**). Determination of senescence-specific cell cycle inhibitors (p16, p21, and p53) in young and DIPS cells. White arrows indicate exemplary positive cells. Magnification × 200, objective aperture 0.35 (**b**). Changes in cyclin B1 and D1 level during DIPS (**c**) quantified with densitometry (**d**). Because the cytostatic effect of drugs is diverse for individual samples, the blots represent results obtained for all tested cultures (Y—young cells; S—senescent/DIPS cells). Results derive from 6–8 independent experiments with HGSOCs obtained from different donors. Flow cytometry was performed using 10 000 cells, whereas the quantification of cell cycle inhibitors was based on the analysis of 500 randomly selected cells per group. The results are expressed as the means ± SEM. *p < 0.05; **p < 0.01; ***p < 0.001 vs. young cells

Flow cytometry analysis of cells stained with propidium iodide revealed that CPT-PCT-treated HGSOCs showed arrested growth in the G2/M phase of the cell cycle, which was reflected by a twofold increase in the fraction of cells identified in this phase with the simultaneous reduction by a half of the fraction of the DNA-replicating cells identified in the S phase (Fig. 2a). This coincided with upregulated expression of p16, p21, and p53 proteins, as evidenced by immunofluorescence. In young cultures, less than 10% of cells displayed expression of the tested cell cycle inhibitors, indicating vigorous proliferation of these cells. When the cells became senescent, the fraction of p16-, p21- and p53-positive cells increased several-fold, in line with the reduced proliferative capacity of these cells (Fig. 2b). The magnitude of this increase in these cell cycle inhibitors was comparable. With changes in cell cycle-promoting cyclins, DIPS cells displayed decreased expression of cyclin B1 and unaltered expression of cyclin D1, as evidenced by western blot analysis. Remarkably, the pattern of the cyclin expression was highly heterogenous, which was plausibly a result of the primary nature of tested cells and several patient-specific and genetic variables (Fig. 2c, d and Additional file 1).

### Analysis of the engagement of telomeres and telomerase

**Fig. 3:**
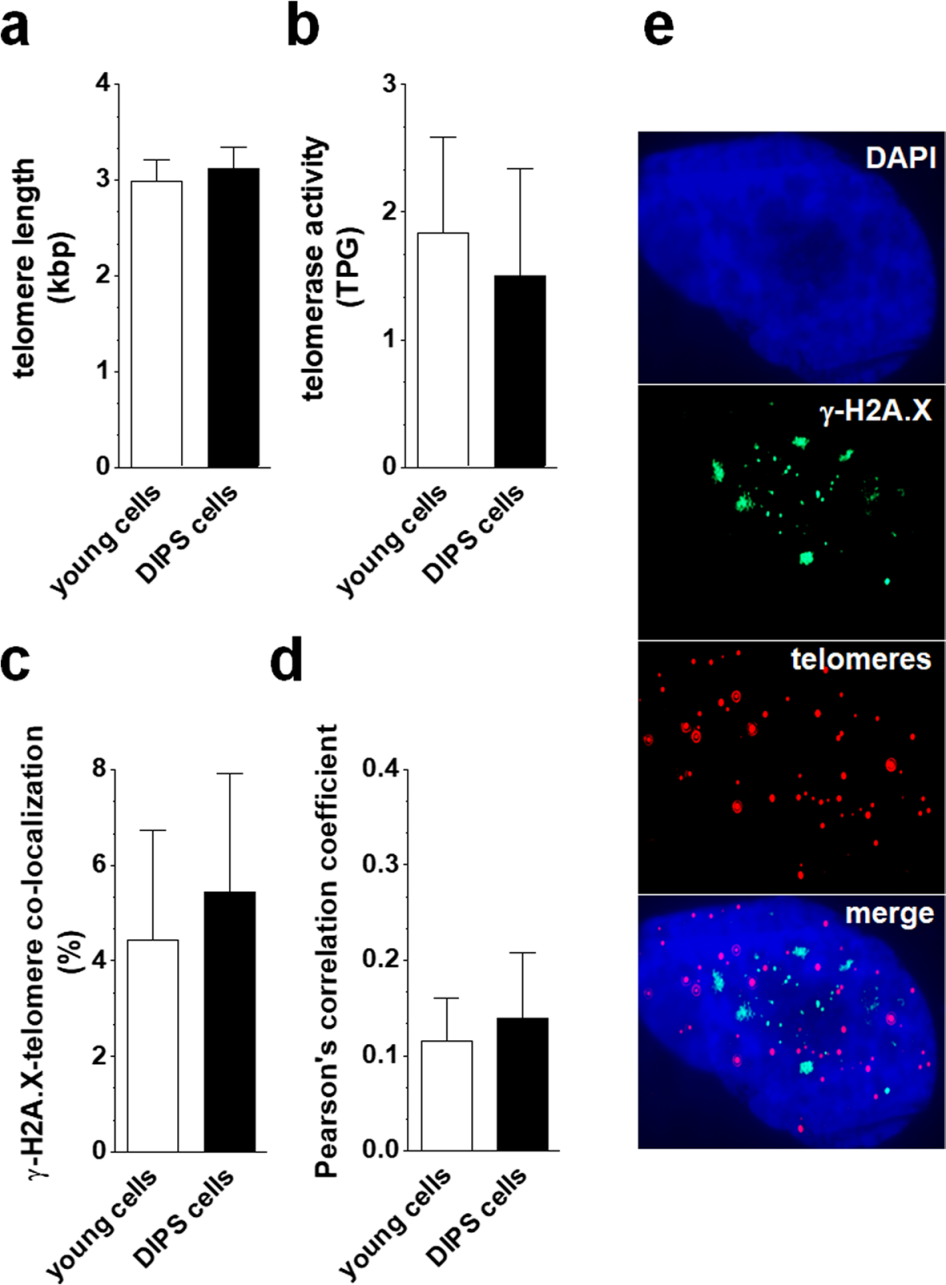
Assessment of the role of telomeres in DIPS of HGSOCs. Changes in telomere length (**a**) and telomerase activity (**b**) during DIPS were analyzed using RT-qPCR. Analysis of the colocalization of histone γ-H2A.X foci (green dots) with telomeric ends (red dots) in young and DIPS cells (**c**, **e**). Determination of Pearson’s correlation coefficient for the colocalization measurements. The image analysis includes identifying areas where green color overlaps with red, yielding yellow. At these locations, DNA damage is present within the telomeres, indicating a telomere-dependent mechanism of senescence. When the two colors do not overlap, i.e., the Pearson’s correlation coefficient is low (far below 1), the DNA damage is located in non-specific regions of the genome, suggesting that cells senesce in a telomere-independent manner (**d**). The results derive from 6–8 experiments with HGSOCs obtained from different donors. The results are presented as the means ± SEM. *TPG* total product generated

Three parameters were quantified to establish the role of telomeres in DIPS of HGSOCs. These were telomere length, telomerase activity, and colocalization of telomeres with γ-H2A.X foci followed by a Pearson’s correlation analysis. Comparative measurements of young cells with DIPS cells showed that telomeres in young cells are relatively short (~ 3 kbp) and that they do not shorten after exposure to CPT + PCT (Fig. 3a). At the same time, a catalytic subunit of telomerase, hTERT, has unaltered activity (Fig. 3b). A search for colocalization of γ-H2A.X foci with telomeres showed that the level of DNA damage within these structures is relatively low in young cells (far below 10%) and does not increase significantly after cell exposure to the drugs (Fig. 3c, e). This observation was consistent with Pearson’s assessment of the correlation between γ-H2A.X foci and telomeres in deconvoluted pictures, which yielded coefficients of 0.15 ± 0.01 and 0.16 ± 0.02 for young and DIPS cells, respectively (Fig. 3d).

### DIPS-associated changes in signaling molecules

**Fig. 4:**
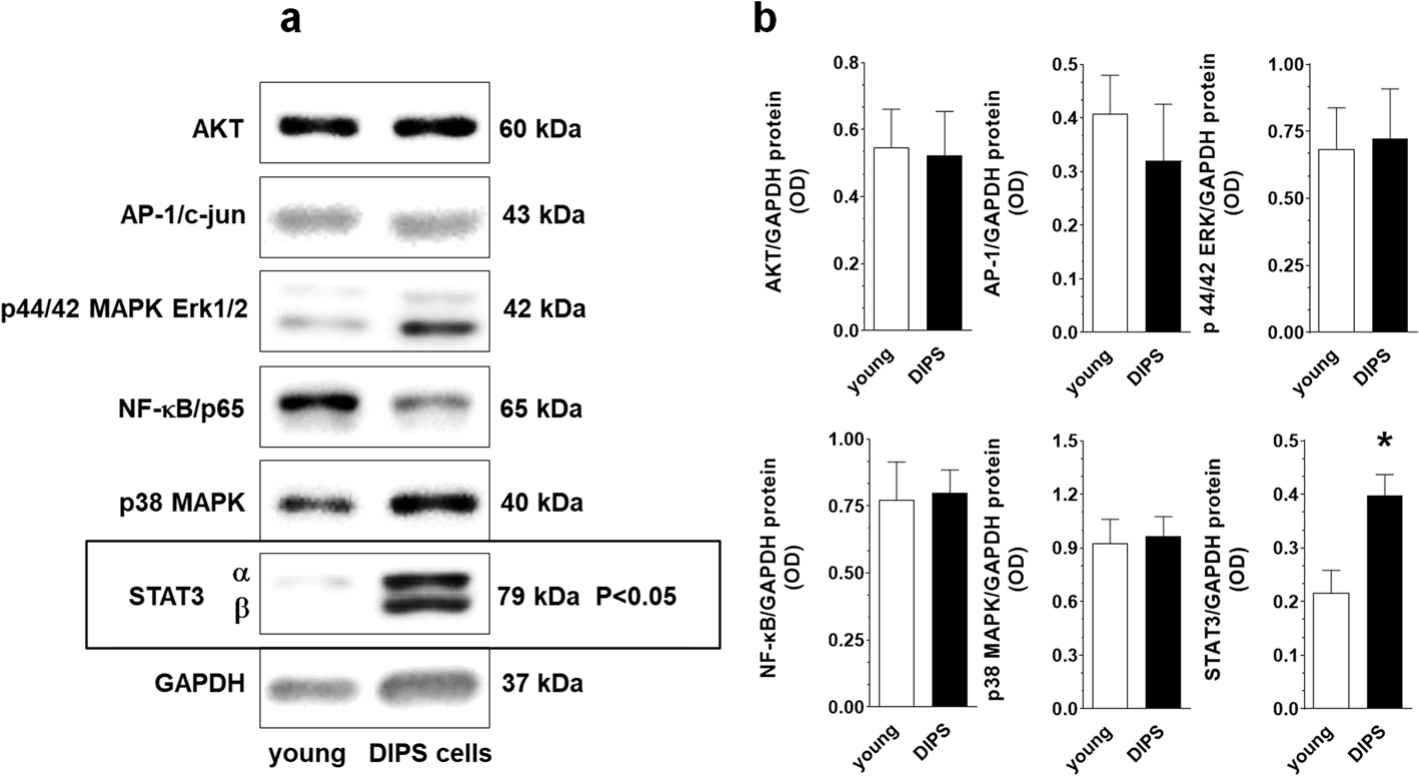
DIPS-associated change**s** in the protein level of kinases and transcription factors arbitrarily selected according to the literature as those being potentially involved in drug-induced cancer cell senescence. Representative bands corresponding to certain signaling molecules analyzed using western blot (**a**) and their densitometric analysis with GAPDH used as the reference protein (**b**). In order to reduce the risk of incorrect observations due to cellular hypertrophy in senescent cells, SDS-PAGE was performed using samples corresponding to the experimentally optimized number of cells (3–5 × 10^4^). The results derive from 5–6 independent experiments on HGSOCs from different patients

Six signaling molecules known to be engaged in cellular senescence, that is, AKT, AP-1, ERK1/2, NF-κB, p38 MAPK, and STAT3, were tested using immunoblotting in young and DIPS cancer cells. The protein levels of the five molecules did not change during senescence, except for STAT3; in the case of this transcription factor, the level of STAT3α markedly increased along with the appearance of the second band representing the STAT3β isoform (Fig. 4a, b and Additional file 1).

### Formation of reactive oxygen species and mitochondrial metabolism during DIPS

**Fig. 5:**
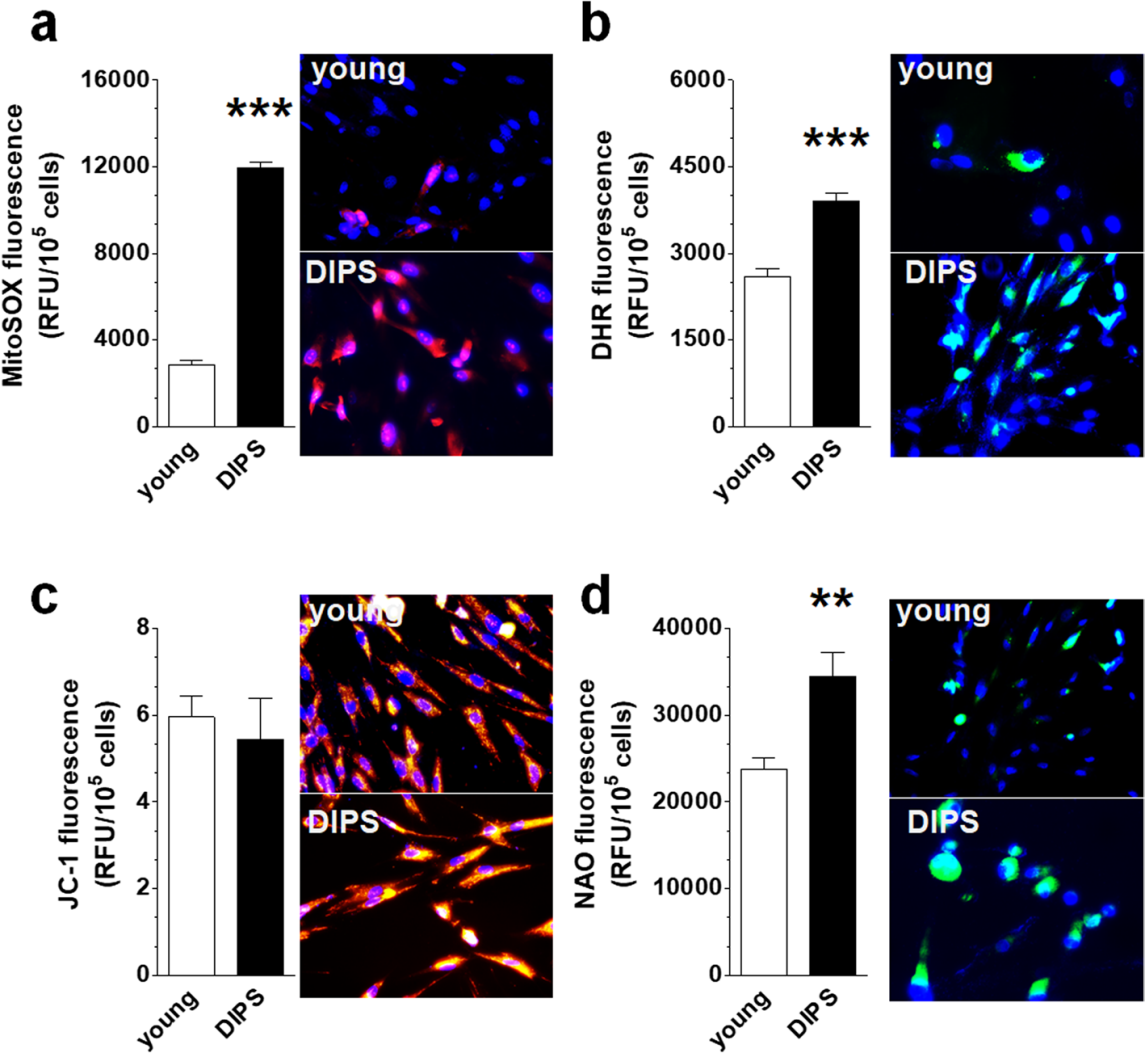
Analysis of oxidative stress determinants during DIPS of HGSOCs. Changes in the production of mitochondrial superoxide (**a**), cellular peroxides (**b**), mitochondrial membrane potential (**c**), and mitochondrial mass (**d**) quantified according to the fluorescence of MitoSOX Red, DHR, JC-1, and NAO, respectively. As per mitochondrial membrane potential measurements, JC-1 accumulates in the functional mitochondria, forming red fluorescent J-aggregates (at 590 nm), while mitochondria de-energization results in the accumulation of green fluorescent monomers (at 535 nm). The rise in the green/red fluorescence intensity ratio indicates a drop of the mitochondrial membrane potential values. The results are based on 8 independent experiments using HGSOCs obtained from different patients. The results are expressed as the means ± SEM. **p < 0.01; ***p < 0.001 vs. young cells. *RFU* relative fluorescence units

DIPS cancer cells produce higher amounts of mitochondrial superoxide and cellular peroxides than young cells, as shown by fluorescent staining of cells with MitoSOX Red (Fig. 5a) and DHR (Fig. 5b), respectively. This effect was accompanied by unaltered values of ΔΨ_m_ potential (JC-1 fluorescence; Fig. 5c) and elevated mitochondrial mass (NAO fluorescence; Fig. 5d).

### DIPS-related changes in mRNA expression and protein release

**Fig. 6:**
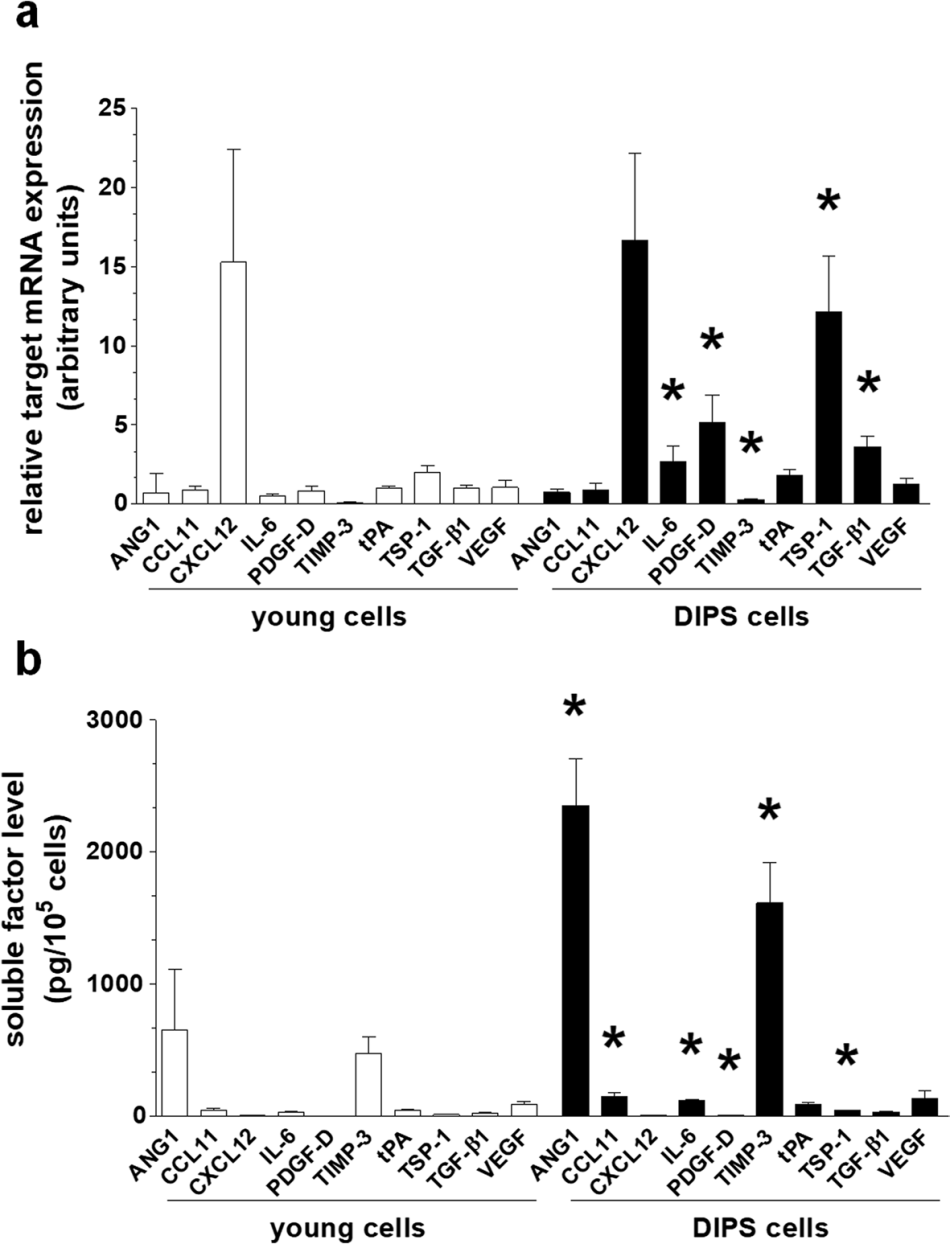
Development of the senescence-associated secretory phenotype (SASP) in DIPS cells. Changes in mRNA of arbitrarily selected targets, as quantified according to RT-qPCR (**a**). Changes in the secretion of respective proteins into the environment (conditioned medium), as quantified by ELISA (**b**). The results are based on 6–8 independent experiments using HGSOCs obtained from different patients. The results are expressed as the means ± SEM. *p < 0.05 vs. young cells

The production of 10 arbitrarily selected proteins involved in various aspects of ovarian cancer cell progression was evaluated at both the mRNA (qPCR; Fig. 6a) and protein levels (ELISA; Fig. 6b). The list included angiopoietin-1 (ANG1), chemokines CCL11 and CXCL12, interleukin 6 (IL-6), platelet-derived growth factor D (PDGF-D), tissue inhibitor of metalloproteinase 3 (TIMP-3), tissue plasminogen activator (tPA), thrombospondin 1 (TSP-1), transforming growth factor β1 (TGF-β1), and vascular endothelial growth factor (VEGF). Analysis of these targets revealed that the production of 4 molecules was upregulated at both the mRNA and protein levels (IL-6, PDGF-D, TIMP-3, and TSP-1), 1 at the mRNA level (TGF-β1), and 2 at the protein level (ANG-1 and CCL11). At the same time, the production of 3 proteins (CXCL12, tPA, and VEGF) did not change in response to DIPS (Fig. 6a, b).

## Discussion

In this study, we provide the first comprehensive evidence that the CPT-PCT mixture, applied to primary HGSOCs in vitro using an experimentally established algorithm and clinically relevant doses [10], triggers the development of a phenotype similar to that of cellular senescence. This finding agrees with the study by Li et al., in which they observed the prosenescence impact of cisplatin. Notably, however, that study was performed on an established ovarian cancer cell line, A2780 [17]. The dynamics of senescence development in primary HGSOCs was similar to that reported in other tumor types [4]. Ovarian cancer cells undergoing drug-induced senescence (DIPS) displayed classical biomarkers of this phenomenon, including the activity of SA-β-Gal [12] and the presence of γ-H2A.X foci [18] (Fig. 1a, b). The recruitment of the second element of the DNA damage response, p53 binding protein 1 (53BP1) [19], did not change in line with γ-H2A.X (Fig. 1a, b), and one may theorize that senescence persistence was enabled by the ineffective repair of DNA double-strand breaks, plausibly due to insufficient 53BP1-controlled nonhomologous end-joining (NHEJ) [20]. Taking into account the knowledge about the effects of platins, both carboplatin [21] and cisplatin [22], on DNA integrity, one cannot exclude that senescence-associated DNA damage in CPT-PCT-treated cells may also include single-strand breaks. At least theoretically, the lack of 53BP1 elevation could also be associated with the fact that 94% of HGSOCs display mutated *P53*, which might translate into decreased reactivity of its binding protein [23].

G2/M growth arrest of HGSOCs, whose senescence was evoked by CPT-PCT (Fig. 2a), is a typical hallmark of cancer cells undergoing DIPS [24] and probably reflects interference of CPT-generated modifications of purines (covalent binding to the N7 sites) with cell replication machinery [25]. The activation of the G2/M checkpoint was also reported in breast cancer cells, whose senescence was provoked by paclitaxel [26], as well as in lung [27] and mammary gland cancer cells [28]. In the latter two cases, this pattern of cell distribution coincided with depressed cyclin B1, which agrees with our findings (Fig. 2c). Intriguingly, G2/M exit from the cell cycle distinguishes DIPS ovarian cancer cells from their counterparts undergoing replicative senescence, in which senescence-associated growth arrest occurred in the G1 phase and involved elevated cyclin D1, whose level in DIPS cells [29] and other cellular models undergoing G2/M phase block [30] was unchanged.

This difference may result from the fact that the replication-dependent senescence of cancer cells involves telomere attrition, which is causatively linked with G1 exit [31]. In the case of DIPS cells, the process is clearly telomere-independent, as it suggests an unchanged telomere length, stable hTERT, and predominantly non-telomeric localization of DNA damage foci (Fig. 3e). Interestingly, the pattern of changes in cell cycle inhibitors, that is, the upregulated expression of p16, p21, and p53 (Fig. 2b), is similar in both types of ovarian cancer cell senescence and is consistent with observations of other cancer [32] and normal cell types rendered senescent by CPT and PCT [33]. The elevation of these inhibitory proteins revealed in DIPS ovarian cancer cells is also in line with the G2/M growth arrest of these cells; recently, similar coincidence has been described in fibroblasts, whose premature senescence was induced by ultraviolet A irradiation [34].

Regarding signaling molecules potentially controlling DIPS in HGSOCs, only signal transducer and activator of transcription 3α (STAT3α [35]) protein increased significantly (Fig. 4a). Notably, in replicatively senescent ovarian cancer cells, the STAT3 level was among the pathways whose level declined. Our observation corresponds, however, with reports in which hyperphosphorylation of STAT3 was found in fibroblasts treated with cisplatin [36] and PCT [37]. Furthermore, the levels of AKT, AP-1, and ERK1/2 in DIPS cells remained unchanged, which also distinguishes them from replicatively senescent ovarian cancer cells, in which the levels of these molecules was elevated [29].

In addition to plausible STAT3 induction by CPT-PCT, some role in this process, additionally facilitated by partial mitochondrial distribution of this factor [38], may also involve reactive oxygen species (ROS) [39]. Indeed, DIPS ovarian cancer cells generate more mitochondrial superoxide (Fig. 5a) and cellular peroxides (Fig. 5b) than young cells, which is generally in line with the pro-oxidative effects of these drugs [28, 40]. At the same time, in contrast to replicatively senescent ovarian cancer cells, this intensified ROS release was not associated with retrograde, compensatory biogenesis of mitochondria, driven by declining values of inner membrane potential (ΔΨ_m_) (Fig. 5c) [29, 41]. Because DIPS cancer cells seem to maintain their ability to generate ATP (unaltered ΔΨ_m_), the more probable scenario is that their elevated mitochondrial content (Fig. 5d) is not the direct reason for increased ROS production but rather a byproduct of CPT-PCT activity. This view supports research on A549 cancer cells in which PCT strongly induces peroxisome proliferator-activated receptor-gamma coactivator (PGC)-1α, the master regulator of mitochondrial biogenesis [42].

The increased level of STAT3 in the presence of stable NF-κB and p38 MAPK, i.e., the leading molecules governing the senescence-associated secretory phenotype (SASP) [43], may also contribute to changes in the DIPS cell transcriptome and secretome. In fibroblasts senesced upon exposure to PCT, the activation of STAT3 was linked with hypersecretion of IL-6 [33, 44], i.e., one of the agents whose mRNA and protein were upregulated in DIPS ovarian cancer cells. According to a recent report, the IL-6-JAK/STAT3 interplay was found to correlate with increased ovarian tumor cell growth and resistance to chemotherapy [45].

In addition to IL-6, DIPS cells also overproduced at the mRNA and/or protein level several agents potentially involved in various aspects of cancer cell progression, in which they resemble, e.g., fibroblasts hypersecreting IL-6 [33] and chemokine (C–C motif) ligand 5 (CCL5) [11] in response to PCT or endothelial cells generating increased amounts of ICAM-1 and IL-8 upon exposure to CPT [46]. The list of ovarian cancer-promoting agents upregulated in DIPS cells includes mediators of cancer cell proliferation (CCL11 – also via STAT3 [47]), migration (TGF-β1 [48]), invasion (PDGF-D [49], TGF-β1 [50]), epithelial-mesenchymal transition (TGF-β1 [51], TSP-1 [52]), and angiogenesis (ANG-1 [53]). This list indicates, in turn, that ovarian cancer cells forced into senescence by CPT-PCT may develop a tumor-promoting phenotype, analogous to the paracrine activity of senescent normal cells, e.g., in the peritoneal mesothelium [54].

## Conclusions

The findings presented in this report improve the understanding of the effect exerted by conventional chemotherapeutics (here, CPT and PCT) on the tumor microenvironment. More precisely, they show that drugs treated as the gold standard in ovarian cancer therapy affect not only the metabolism and behavior of normal (peritoneal) cells [8] but may also, paradoxically, contribute to the development of cancer-promoting features in ovarian cancer cells by provoking their premature senescence. Because knowledge about the biological and clinical outcomes of ovarian cancer cell senescence is still very limited, a detailed comparative analysis of the plausible cancer-promoting effects of replicatively senescent and DIPS cancer cells using both in vitro and in vivo models is urgently required. To make the whole picture of the discussed processes clearer and more reliable, a separate group of these tests should include cells from patients in whom CPT and PCT were administered in vivo as elements of their therapy. Another critical issue that has to be additionally verified is the concentration of drugs and time of their administration. The results of this study were based on doses matching as much as possible the in vivo conditions. The time of drug activity is however more complex because in vivo it is a sum of their actual delivery and further activity in tissues associated with patient-specific pharmacokinetics, whereas in vitro it was fixed at the value of 72 h.

## Supplementary Information

**Additional file 1:**: Original uncropped and unprocessed immunoblots for cyclins and signaling molecules.

## Data Availability

All data generated or analyzed during this study are included in this published article and its additional information files. Remaining materials, including microphotographs, are in the possession of the corresponding author and will be shared upon request.

## Abbreviations

53BP1: P53-binding protein 1
CPT: Carboplatin
DIPS: Drug-induced premature senescence
HGSOCs: High-grade serous ovarian cancer cells
PCT: Paclitaxel
SA-β-Gal: Senescence-associated β-galactosidase
SASP: Senescence-associated secretory phenotype
STAT3: Signal transducer and activator of transcription 3
