# Supplementary material for: Primary high-grade serous ovarian cancer cells are sensitive to senescence induced by carboplatin and paclitaxel in vitro
Source: Cell Mol Biol Lett. 2021 Oct 21;26:44. doi: 10.1186/s11658-021-00287-4 (PMC8532320; doi:10.1186/s11658-021-00287-4)
Supplement: Supplementary file 1 — Additional file 1:: Original uncropped and unprocessed immunoblots for cyclins and signaling molecules. [file 11658_2021_287_MOESM1_ESM.pdf]

Original, uncropped and unprocessed blots for cyclin B1, D1, and GAPDH

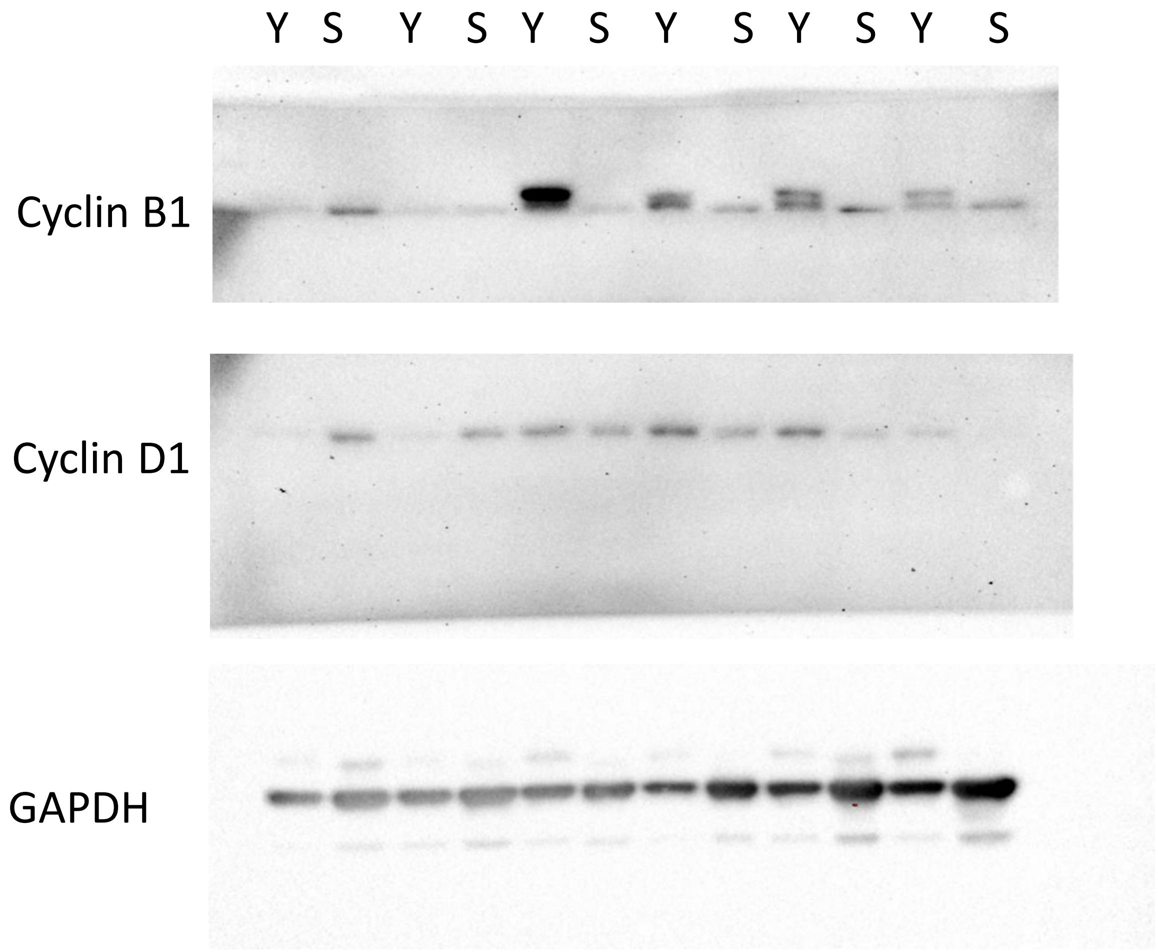

Y – young
S - senescent

Original, uncropped and unprocessed blots for signaling molecules

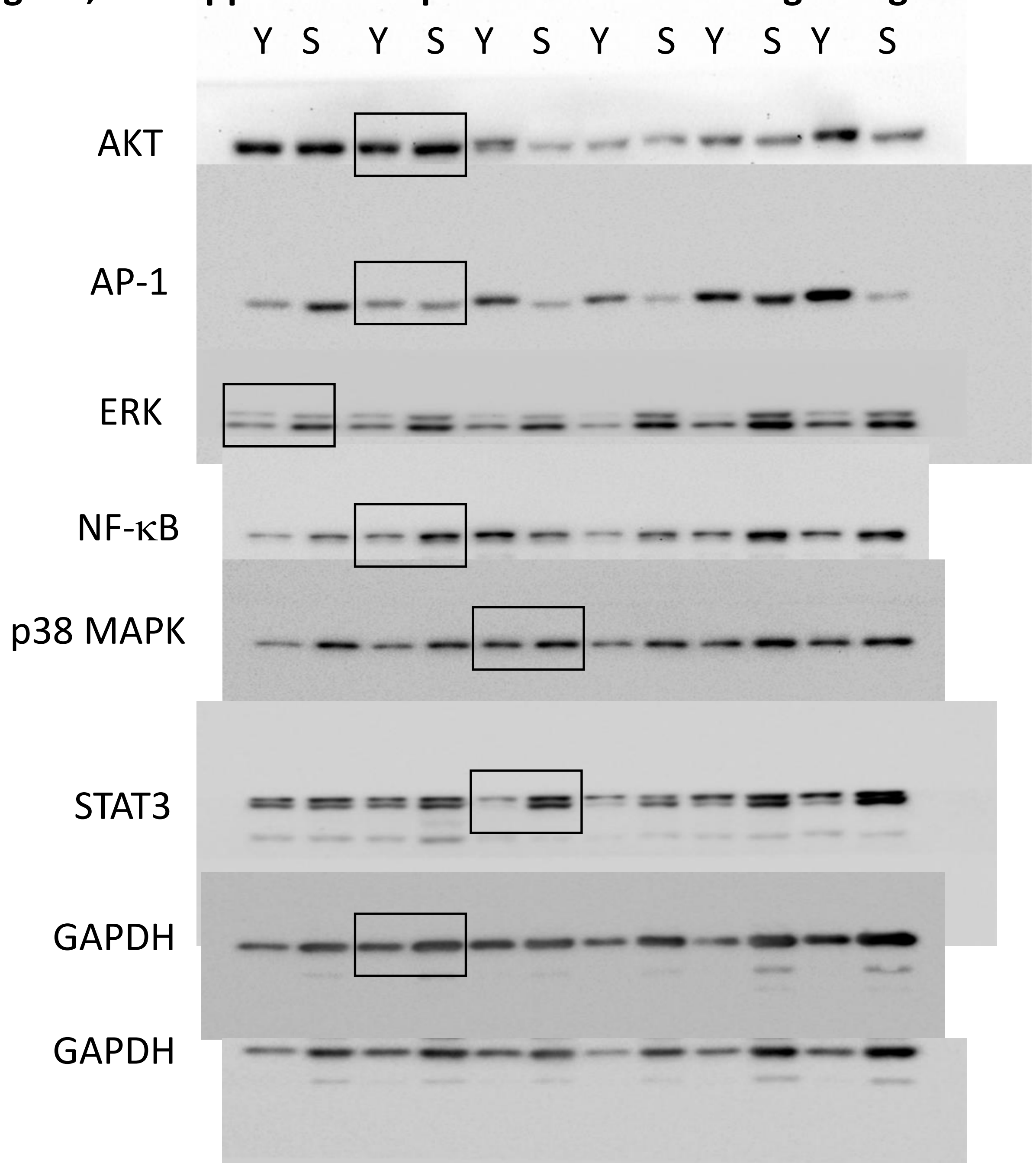

Y – young
S - senescent
